# Adjudin-loaded redox-sensitive paclitaxel-prodrug micelles for overcoming multidrug resistance with efficient targeted Colon cancer therapy

**DOI:** 10.1080/10717544.2020.1797245

**Published:** 2020-07-24

**Authors:** Deli Chen, Sitang Ge, Lugen Zuo, Shuanhu Wang, Mulin Liu, Shiqing Li

**Affiliations:** Department of Gastrointestinal Surgery, The First Affiliated Hospital of Bengbu Medical College, Bengbu, China

**Keywords:** Multidrug resistance, co-delivery, mitochondria-inhibition, adjudin

## Abstract

Multidrug resistance (MDR) is the primary cause for the failure of chemotherapy in the treatment of colon cancer. Recent research has indicated that the combination of a chemotherapeutic agent and a mitochondrial inhibitor might represent a promising strategy to help overcome MDR. However, for this approach to be clinically effective, it is important that the two drugs can be actively and simultaneously delivered into tumor cells at an optimal ratio and completely released drug within cells. To address these challenges, we designed and prepared a folate receptor-targeted and redox-responsive drug delivery system (FA-*ss*-P/A) that was able to co-deliver paclitaxel (PTX) and adjudin (ADD) to reverse colon cancer MDR. The PTX prodrug was obtained by conjugating PTX to dextrin via a disulfide-linkage. Then, folic acid (FA) was modified on the PTX prodrug. Finally, ADD, a mitochondrial inhibitor, was encapsulated in the PTX prodrug-formed micelles. A series of in vitro and in vivo experiments subsequently demonstrated that FA-*ss*-P/A can effectively reverse MDR by increasing cell uptake, inhibiting PTX efflux, and improving drug release.

## 1. Introduction

Colon cancer is one of the most common forms of cancer, and the third primary cause of cancer-associated mortality globally (O'Brien et al., 2007; Jemal et al., 2010). Although considerable effort has been made to identify effective treatments for colon cancer, chemotherapy remains the predominant form of treatment for advanced colon cancer; however, the curative effect of this form of treatment is significantly reduced by multidrug resistance (MDR) (Riganti et al., 2005; Hasani-Sadrabadi et al., 2016). This is a phenomenon in which cancer cells develop resistance to the effects of numerous cytotoxic agents (Wang et al., 2015). Several mechanisms have been demonstrated to be associated with MDR; these involve energy-dependent ATP-binding cassette transporters (ABC transporters) such as P-glycoprotein (P-gp) and multidrug resistance protein 1 (MRP1) (Gottesman & Pastan, 1993; Szakács et al., 2006; Xiaopin et al., 2013; Wang et al., 2016; Muddineti et al., 2017). These proteins can increase drug efflux and reduce drug uptake, resulting in an intracellular level of drugs that is not sufficient to destroy cancer cells (Yang et al., 2014; Gupta et al., 2017; Li et al., 2017). At present, there are two main strategies used to combat MDR: nanomedicine, and the co-administration of chemotherapeutic agents and efflux pump regulators (Che et al., 2019). Although nanomedicine can bypass ABC transporters via the mechanism of endocytosis, drugs that are released intracellularly may still be at risk of being removed by ABC transporters (Wei et al., 2016; Che et al., 2019). Furthermore, the combined use of therapeutic drugs and efflux regulators is severely restricted owing to the inconsistent biodistribution and pharmacokinetics of chemotherapeutic agents and efflux pump regulators, and the numerous side effects associated with the latter (Wei et al. 2016; Che et al., 2019). Remarkably, the co-delivery of chemotherapeutic agents and efflux pump regulators via nanoplatforms may lead to significant improvements in their ability to treat MDR by harnessing the advantages of ABC transporter inhibition and nanomedicine (Shi et al., 2015).

Adjudin (ADD), an analogue of lonidamine, is a powerful inhibitor of mitochondrial function (Xie et al., 2013). ADD is able to induce mitochondrial dysfunction, resulting in the severe disruption of energy supply and inhibition of the ATP-dependent efflux transporter, P-gp, ultimately reversing MDR (Li et al., 2015; Qi et al., 2018; Wang et al., 2019). Several previous studies have demonstrated that the co-delivery of ADD and doxorubicin (DOX) effectively reverses MDR in breast cancer (Li et al., 2015; Qi et al., 2018; Wang et al., 2019). It has also been reported that the co-delivery of two drugs, at an optimized ratio, and with rapidly and completely intracellular drug release, can achieve best therapeutic effects (Xiaopin et al., 2013; Yang et al., 2019). A previous report solved the problem of co-delivering ADD and DOX at an optimal ratio by utilizing a pH-sensitive prodrug nanosystem (Wang et al., 2019). However, the total amount of DOX that was released within 72 h at pH 5.0 was lower than 30%. Thus, by developing an appropriate nanocarrier for the co-delivery of chemotherapeutic drugs and ADD, with an optimized ratio and maximal levels of intracellular drug release, may significantly improve the combination effect.

Dextran (DEX), a natural hydrophilic polysaccharide, has good biocompatibility and biodegradability. DEX has also been used as a plasma volume extender to enhance peripheral blood flow for many years (Li et al., 2017). More importantly, DEX exhibited more stability and less tend to nonspecific protein adsorption as drug carrier than PEG (Chechushkov et al., 2016). Therefore, DEX was provided as one of the most ideal and safe biomedical material.

**Scheme 1.**
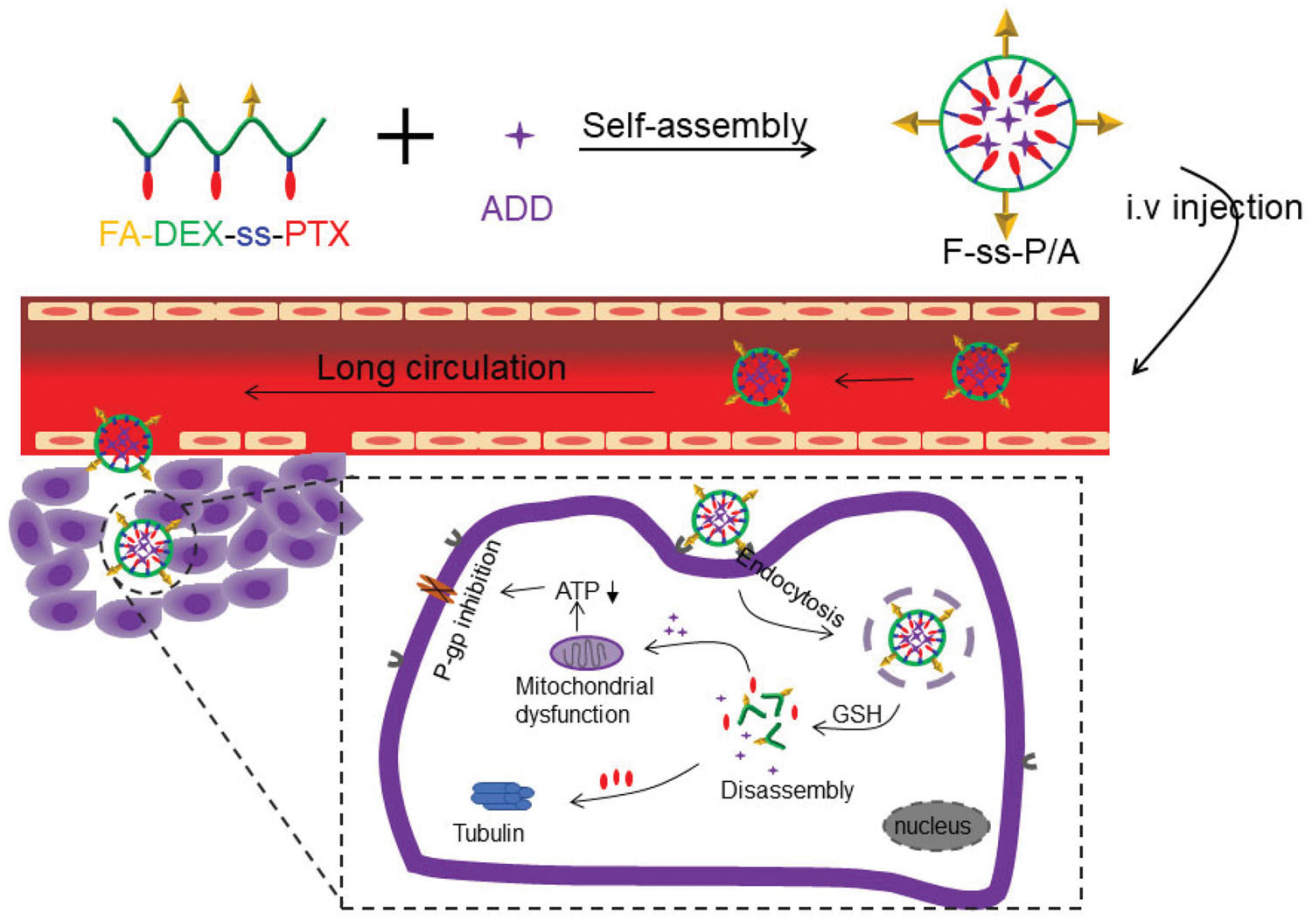
Schematic illustration of the self-assembly of FA-*ss*-P/A micelles, colon cancer cell-targeted drug delivery, intracellular GSH-responsive drug release, and overcome MDR mechanism.

To address these problems, we designed a redox-responsive paclitaxel (PTX) prodrug polymeric micelle able to co-deliver ADD in order to combat MDR in patients with colon cancer (Scheme 1). A number of studies have demonstrated that redox-responsive prodrugs can achieve completely intracellular drug release owing to the high levels of glutathione (GSH) in cancer cells (Yin et al., 2015). In our study, we conjugated PTX to DEX via a disulfide linkage to create the PTX prodrug. Moreover, to increase the accumulation of drugs at the tumor site, we modified the folic acid (FA) on the surface of PTX prodrug micelles to act as the tumor-active targeting ligand (Cheng et al., 2017; Zhong et al., 2017). Finally, ADD was encapsulated in the hydrophobic core of the PTX prodrug micelles via van der Waals forces; this created the final delivery system, which we named F-*ss*-P/A. We hypothesized that this co-delivery system could effectively overcome MDR in colon patients, by combining tumor active-targeting ability, intracellular drug-release capability, and the inhibition of mitochondrial function.

## 2. Materials and experiments

### 2.1. Synthesis of DTPA-PTX

DTPA-PTX was synthesized as described previously, but with some modifications (Li et al., 2014). In brief, PTX (400.0 mg, 0.468 mmol), DTPA (122.9 mg, 0.585 mmol), EDC (123.6 mg, 0.6435 mmol), and DMAP (74.0 mg, 0.6435 mmol), were dissolved in 60 mL of dry DMSO and stirred at 37 °C for 48 h under a dry nitrogen atmosphere. At the end of the experiment, the solution was precipitated with 600 mL of 0.1 M dilute hydrochloric acid. Subsequently, the precipitation was washed three times with 0.1 M dilute hydrochloric acid and distilled water. The final product was obtained after drying under vacuum for 48 h (yield: 63.2%).

As a control, we also prepared the non-redox-sensitive PTX prodrug, glutaric anhydride-paclitaxel (GA-PTX). In brief, PTX (400.0 mg, 0.468 mmol), GA (58.7 mg, 0.515 mmol), and DMAP (75.4 mg, 0.618 mmol) were dissolved in 50 mL of dry DMSO and stirred at 37 °C for 18 h under a dry nitrogen atmosphere. At the end of the experiment, the solution was precipitated with 600 mL of 0.1 M dilute hydrochloric acid. Subsequently, the precipitation was washed three times with 0.1 M dilute hydrochloric acid and distilled water. The final product was obtained after drying under a vacuum for 48 h (yield: 65.4%).

### 2.2. Synthesis of DEX-*ss*-PTX

DEX-*ss*-PTX was obtained by conjugating DTPA-PTX to DEX via an esterification reaction. In brief, DEX (1.0 g, 0.085 mmol), DTPA-PTX (890.8 mg, 0.85 mmol), EDC (195.8 mg, 1.02 mmol), and DMAP (124.4 mg, 1.02 mmol) were dissolved in dry DMSO and stirred at 37 °C for 24 h under a dry nitrogen atmosphere. Then, the solution was dialyzed (MWCO: 3,500 Da) against DMSO to remove the unreacted DTPA-PTX, and then dialyzed against distilled water to remove DMSO. The final product, DEX-*ss*-PTX, was obtained by freeze-drying (yield: 66.5%).

We also prepared DEX-*cc*-PTX as controls by conjugating GA-PTX to DEX using the same methodology (yield: 59.3%).

### 2.3. Synthesis of FA-DEX-*ss*-PTX

FA-DEX-*ss*-PTX was synthesized using methodology described previously (Hao et al., 2013). Typically, DEX-*ss*-PTX (3,000 mg, about 0.1 mmol), FA (220.5 mg, 0.5 mmol), EDC (115.2 mg, 0.6 mmol), and DMAP (73.2 mg, 0.6 mmol), were dissolved in 100 mL of dry DMSO and stirred at 37 °C for 24 h under a dry nitrogen atmosphere. Then, the solution was dialyzed (molecular weight cutoff, MWCO: 3,500 Da) against phosphate buffer (PBS, pH 7.4, 10 mM) containing 0.15 M NaCl to remove the unreacted FA, and then dialyzed against distilled water. The final product, FA-DEX-*ss*-PTX, was obtained by freeze-drying (yield: 74.1%).

The PTX content of FA-DEX-*ss*-PTX, DEX-*ss*-PTX, and DEX-*cc*-PTX was determined using a UV-light spectrometer, and calculated according to Equation (1).

(1) $$\text{PTX content }=\;\frac{\mathrm{weight}\;of\;\mathrm{PTX}}{\mathrm{weight}\;of\;\mathrm{the}\;\mathrm{whole}\;\mathrm{polymer}}\;\times 100\%$$

### 2.4. Determination of critical micelle concentration (CMC)

FA-DEX-*ss*-PTX, DEX-*ss*-PTX, and DEX-*cc*-PTX were first dissolved to the required concentration in PBS. Then, we added Nile Red solution (dissolved in DMSO) to a final concentration of 6 × 10^−7 ^M. The fluorescence of this solution was then measured using a fluorescence spectrometer (excitation light: 557 nm; emitted light: 601 nm). The CMC was then determined by extending the linear fluorescence intensity of both the high and low regions of concentration.

### 2.5. Micelle preparation

All drug-loaded micelles were prepared using the nanoprecipitation method. For example, we prepared PTX micelles that were co-loaded with ADD by combining **A**DD to **FA**-DEX-***ss***-**P**TX; this formulation was referred to as FA-*ss*-P/A. In brief, 15 mg of FA-DEX-*ss*-PTX and 1 mg of ADD were dissolved together in 1 mL of DMSO and stirred vigorously for 2 h under a dry nitrogen atmosphere. Then, the mixture was added dropwise to 10 mL of PBS with vigorous stirring. After 30 min of stirring, the solution was transferred into a dialysis bag (MWCO: 3,500 Da) and dialyzed against PBS at 4 °C for 8 h to remove the DMSO. Finally, we removed the unloaded ADD in the micellar solution by filtering through a 0.45 µm microporous membrane to generate the FA-*ss*-P/A.

The drug loading content (DLC) and encapsulation efficiency (DEE) were calculated according to Equations (2) and (3).

(2) $$\mathrm{DLC}\;(\mathrm{wt}\%)\;=\frac{\text{weight of the drug in the micelles}}{\text{weight of the whole micelles}}\;\times 100\%$$

(3) $$\mathrm{DEE}\;(\mathrm{wt}\%)\;=\frac{\text{weight of the drug in the micelles}}{\text{weight of feed drug}}\;\times 100\%$$

### 2.6. GSH-triggered drug release and micelle disassembly

Next, we used a dialysis method to test the release of PTX and ADD from FA-*ss*-P/A micelles. Typically, a freshly prepared solution of FA-*ss*-P/A micelles was transferred to a dialysis bag (MWCO: 3,500 Da). Then, the dialysis bag was immersed in release media (PBS, pH 7.4 containing 0.1% w/v Tween 80, and various concentrations of GSH) at 37 °C with shaking at 150 rpm. At specific intervals, 200 µL of buffer solution was collected and replaced with 200 µL of fresh release medium. The amounts of PTX and ADD released at each time point was then determined by an HPLC method, as described above.

To investigate the disassembly of micelles, as triggered by GSH, we investigated the change in size of *ss*-P and *cc*-P micelles under various concentrations of GSH, using dynamic light scattering (DLS) and transmission electron microscope (TEM). In brief, 1 mL of micellar solution (in which the concentration of micelles was 2 mg/mL), in different concentrations of GSH (0, 10 µM, and 10 mM, respectively), was incubated at 37 °C with shaking at 100 rpm. We then monitored changes in the size distribution of these samples by DLS at predetermined time points. We also investigated the morphology of these micelles by TEM under the 10 mM GSH condition.

### 2.7. The detection of P-gp expression

The expression levels of P-gp were determined by western blot (WB) assays. Cell samples were prepared in standard RIPA buffer (Beyotime Biotechnology, China) containing protease inhibitor (Thermo Fisher Scientific, USA). Protein concentrations were measured with a bicinchoninic acid (BCA) Kit (Beyotime Biotechnology, China), in accordance with the manufacturer’s protocol. For each sample, an equal amount of protein was then separated by sodium dodecyl sulfate-polyacrylamide gel electrophoresis (SDS-PAGE). After electrophoresis, the proteins were transferred to a polyvinylidene fluoride (PVDF) membrane, blocked with nonfat skimmed milk, and incubated with primary antibodies (anti-P-gp and anti-actin, Abcam, UK). The membranes were washed three times with TBST and then incubated with horseradish peroxidase-conjugated secondary antibody. Finally, target protein expression was visualized using ECL Plus Western Blotting detection reagents (Thermo Fisher Scientific, USA).

### 2.8. *In vitro* cytotoxicity assay

Standard MTT assays were used to determine the cytotoxicity of different formulations against HCT-8 and HCT-8/PTX cells. Typically, cells were seeded in 96-well plates at a density of 5,000 cells/well, and incubated for 24 h in culture conditions. Subsequently, cells were treated with different formulations for 48 h in culture. At the end of the treatment period, 10 µL of MTT (5 mg/mL) solution was added into each well and incubated for 4 h. Finally, 100 µL of DMSO was added into each well to dissolve the internalized purple formazan crystals, and the absorbance of each well was detected by a BioRed microplate reader (MK3, Thermo, USA) with a detection wavelength of 490 nm. Relative cell viability was then calculated using Equation (4), in which As and Ac represent the absorbance in the absence and presence of the drug, respectively. Ab represents the absorbance of the culture medium.

(4) $$\text{Cell viability }(\%)\;=\frac{As-Ab}{Ac-Ab}\times 100$$

### 2.9. Cellular uptake assays and FA enhancement

Coumarin-6 (C6) was chosen as the probe to investigate the cellular uptake behavior of HCT-8/PTX cells. The method used to prepare C6 labeled FA-*ss*-P/A and *ss*-P/A micelles was the same as described previously. The DLC of C6 in FA-*ss*-P/A and *ss*-P/A was 3.2% and 2.8%, respectively. Cellular uptake was observed using confocal laser scanning microscopy (CLSM, Zeiss, Germany) and flow cytometry (FCM, FACSCalibur, BD Biosciences, USA). For CLSM analysis, cells were seeded into a small confocal dish at density of 5 × 10^5^ cells per well and incubated overnight. Subsequently, the cells were first treated with free FA (2 mM) or not for 2 h; then, the C6 labeled FA-*ss*-P/A or *ss*-P/A micelles were added and incubated for different time periods. After incubation, the cells were washed with PBS, fixed with 4% paraformaldehyde, stained with DAPI, and then observed by CLSM.

For FCM analysis, cells were seeded into a six-well plate at density of 5 × 10^5^ cells per well and incubated overnight. Subsequently, the cells were first treated with free FA (2 mM) or not for 2 h; then, the C6 labeled FA-*ss*-P/A or *ss*-P/A micelles were added and incubated for different time periods. At the end of the culture period, the cells were washed with PBS and harvested, resuspended in 400 µL of PBS and finally analyzed by FCM.

### 2.10. PTX efflux analysis

HCT-8 and HCT-8/PTX cells were seeded in 6-well plates at a density of 5 × 10^5^ cells per well and incubated overnight. Then, the cells were treated with PTX, PTX + ADD, FA-*ss*-P, and FA-*ss*-P/A for 4 h (the concentrations of PTX and ADD were each fixed at 20 µg/mL). Subsequently, the cells were further incubated with drug-free medium for 1, 2, and 4 h at 37 °C. At the end of the incubation, cells were collected and lysed with RIPA buffer. Then, 100 µL of cell lysates was extracted with 2 mL of diethyl ether and centrifuged at 10,000 rpm for 10 min at 4 °C. The upper organic layer was transferred to clean tubes and dried under nitrogen gas. Finally, the products were dissolved with methanol and analyzed by HPLC. Prior to HPLC analysis, samples were first treated with an excess of GSH.

### 2.11. Investigating the mechanisms responsible for the reversal of MDR

#### 2.11.1. Determination of mitochondrial membrane potential

We detected the mitochondrial membrane potential with a JC-1 fluorescence probe. HCT-8/PTX cells were seeded into 6-well plates at a density of 5 × 10^5^ cells per well and cultured overnight. The cells were then treated with different drug formulations for 12 h at a PTX concentration of 5 μg/mL. At the end of the incubation period, the cells were washed, trypsinized, collected, and analyzed with a JC-1 mitochondrial transmembrane potential assay kit in accordance with the manufacturer’s protocol. Subsequently, the cells were analyzed by FCM.

#### 2.11.2. Determination of ATP content

HCT-8/PTX cells were seeded into 6-well plates at a density of 5 × 10^5^ cells per well and cultured overnight. The cells were then treated with different drug formulations for 12 h at a PTX concentration of 5 μg/mL. At the end of the incubation period, the cells were washed, trypsinized, collected, and treated with an ATP assay kit according to the manufacturer’s protocol, and then analyzed by FCM.

### 2.12. Pharmaceutics and biodistribution

We created an HCT-8/PTX tumor-bearing mouse model by subcutaneously injecting the right flank of each mouse with HCT-8/PTX cells (6 × 10^6^ cells per mouse). When tumors reached a size of approximately 100 mm^3^, the mice were injected intravenously (*i.v*.) through the tail vein with Taxol, or FA-*ss*-P/A micelles, at an equivalent dose of 6 mg/kg of PTX. Subsequently, three mice were sacrificed at 5 min, 30 min, 1 h, 2 h, 4 h, 8 h, 12 h, and 24 h, after injection. Upon sacrifice, we harvested samples of plasma, liver, kidney, spleen, lung, heart, and tumor. These were first rinsed with PBS, frozen in liquid nitrogen, and then stored at −80 °C to await drug analysis.

For pharmaceutical analysis, 100 µL of plasma was extracted with 2 mL of diethyl ether and centrifuged at 10,000 rpm for 10 min at 4 °C. The upper organic layer was then transferred to a clean tube and dried under nitrogen gas. Finally, the products were dissolved with methanol and analyzed by HPLC. For biodistribution analysis, 200 µL of tissue homogenate was added to 200 µL acetonitrile, and vortexed for 15 min. Subsequently, the mixture was centrifuged at 10,000 for 15 min at 4 °C and 20 µL of supernatant was subjected to HPLC analysis. Prior to HPLC analysis, samples were first treated with an excess of GSH.

### 2.13. *In vivo* antitumor activities

HCT-8/PTX xenograft tumor model mice were randomly divided into seven treatment groups (saline, Taxol, Taxol + ADD, *ss*-P, *cc*-P, FA-*ss*-P, and FA-*ss*-P/A micelles), with six animals per group. Mice were then administered 6 mg/kg of PTX on day 0, 3, and 6, three times a day. The tumor volume (V) was measured every other day and calculated in accordance with Equation (5), in which ‘*a*’ and ‘*b*’ represented the length and the width of the tumor, respectively.

(5) $$\text{V}=\left(a\times b^{2}\right)/2$$

All mice were sacrificed on day 21. Tumors were then harvested, weighed and stained with hematoxylin and eosin for hi$V=\left(a\times b^{2}\right)/2$ stological analysis.

## 3. Results and discussion

### 3.1. Synthesis and characterization of FA-DEX-*ss*-PTX

**Figure 1.**
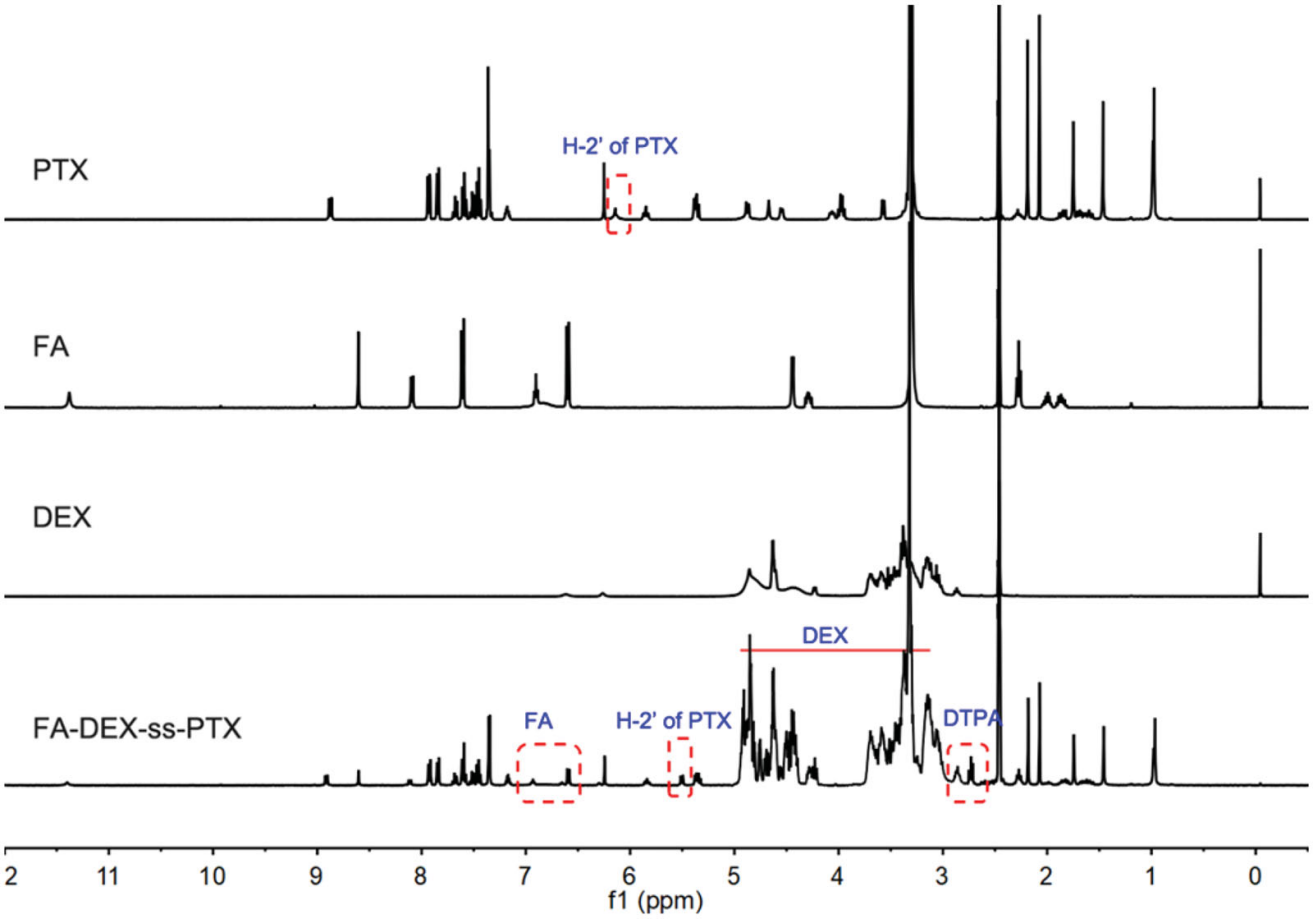
The ^1^H NMR spectrum of PTX, FA, DEX, and FA-DEX-*ss*-PTX in DMSO-*d6*.

**Scheme 2.**
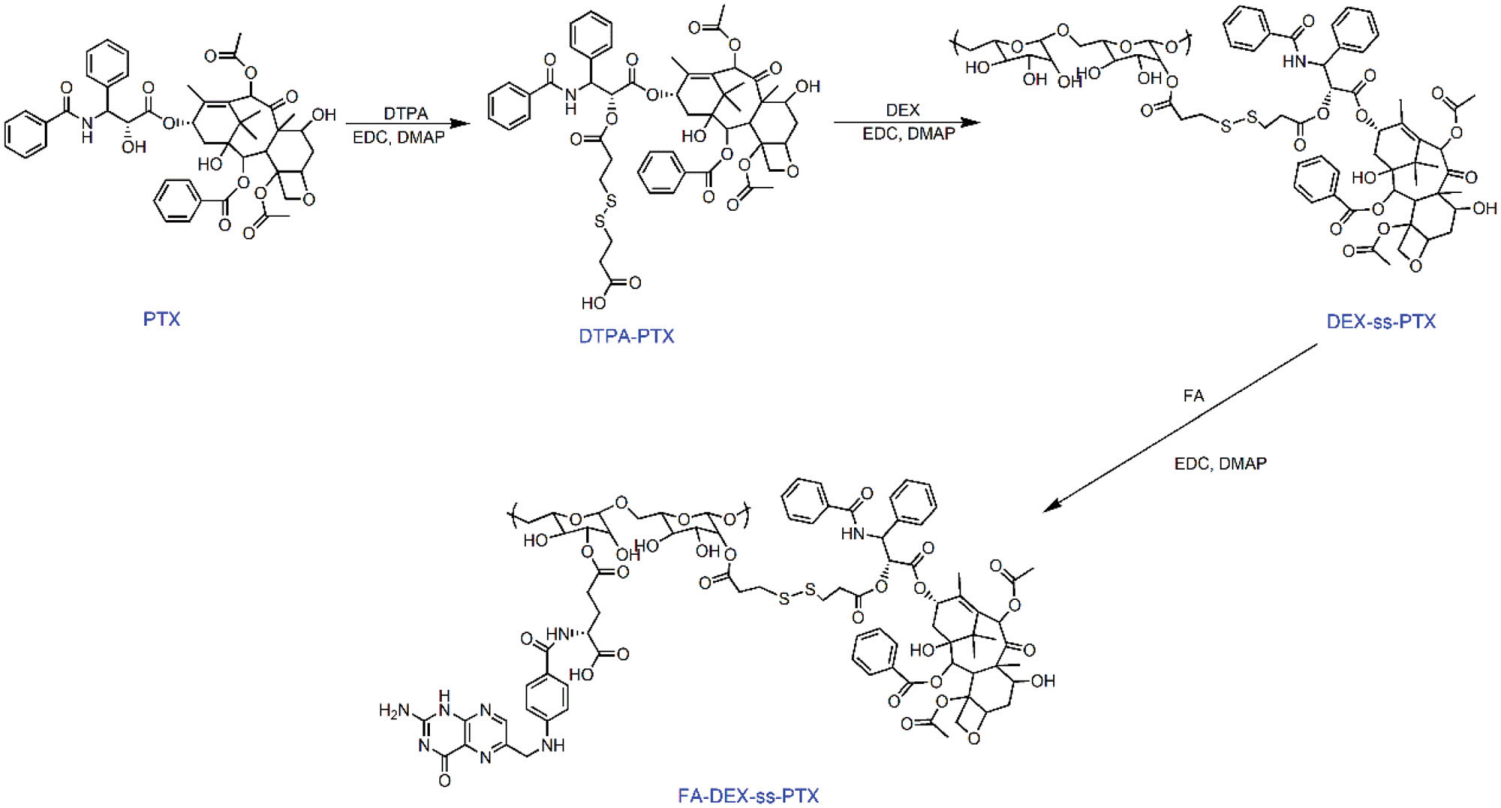
Synthesis route of FA-DEX-*ss*-PTX

In this study, we developed a reduction-sensitive PTX prodrug, FA-DEX-*ss*-PTX. The synthesis of FA-DEX-*ss*-PTX was divided into three steps (Scheme 2). DTPA-PTX was first prepared and characterized by ^1^H NMR and MS (Supplementary Figures S1 and S2). The H-2′ of PTX shifted from 4.8 ppm (free PTX) to 5.6 ppm (conjugated PTX), thus demonstrating the successful preparation of DTPA-PTX. Then, DTPA-PTX was conjugated to DEX to create the DEX-*ss*-PTX, and its structure was verified by ^1^H NMR (Supplementary Figure S3). Finally, FA-DEX-*ss*-PTX was synthesized by conjugating FA to the side chain of DEX-*ss*-PTX. The structure of FA-DEX-*ss*-PTX was subsequently confirmed by ^1^H NMR. As shown in Figure 1, the peaks at 3.0 ppm to 5.0 ppm relate to the DEX, the peaks at 7.4 ppm to 8.0 ppm relate to PTX, the peaks at 2.5 ppm to 2.8 ppm relate to DTPA, and the peaks at 6.6 ppm and 6.9 ppm relate to FA. The appearance of these peaks demonstrated that FA-DEX-*ss*-PTX had been successfully synthesized. The control PTX prodrugs, FA-DEX-*cc*-PTX (Supplementary Figure S4), were also prepared by the same method, and confirmed by ^1^H NMR. The FA graft degree in FA-DEX-*ss*-PTX and FA-DEX-*cc*-PTX is about 4.9% and 5.4%, calculated by the peak area ratio of 6.58 ppm (2H in phenylene) to 4.22 ppm (1H of anomeric carbon in glucose unit). HPLC was further performed to confirm the FA-DEX-*ss*-PTX. As shown in Supplementary Figure S5, PTX and FA-DEX-*ss*-PTX exhibited monodisperse peak at elution time of 8.75 and 4.42 min, respectively. When FA-DEX-*ss*-PTX was incubated with GSH for 2 h, the peak of FA-DEX-*ss*-PTX apparently lowered, a new peak belonged to PTX appeared at elution of 8.75 min, suggesting rapid release of active PTX through cleavage of the disulfide linkage.

### 3.2. Preparation and characterization of drug-loaded micelles

Prior to preparing drug-loaded micelles, we used MTT to determine the optimal combination ratio between PTX and ADD. As shown in Supplementary Figure S6, when the molar ratio of PTX and ADD was fixed at 1: 2, the IC_50_ value was the lowest in comparison with that of the other groups. Thus, the molar ratio of PTX and ADD was fixed at 1: 2 for all drug formulations used in subsequent experiments.

**Figure 2.**
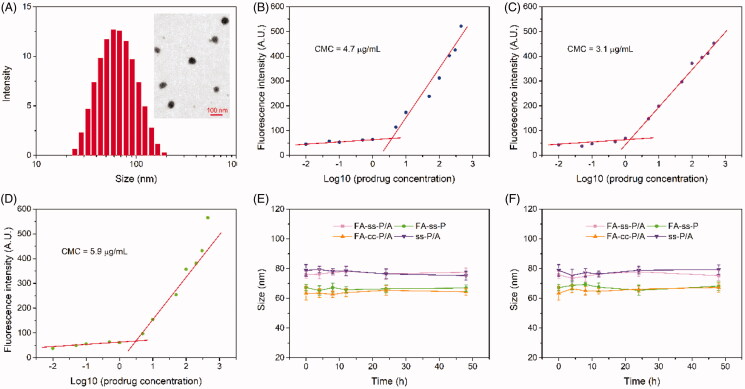
(A) Size distribution and TEM image of FA-*ss*-P/A. (B-D) CMC spectrum of FA-DEX-*ss*-PTX (B), FA-DEX-*cc*-PTX (C), and DEX-*ss*-PTX (D). (E, F) Changes in size for FA-*ss*-P/A, FA-*ss*-P, FA-*cc*-P/A, and *ss*-P/A, after incubation with PBS (E), and PBS with 10% FBS (F). Data shown as mean ± SD, *n* = 3.

All drug-loaded micelles were prepared by a nanoprecipitation method. The PTX and ADD co-loaded micelle with redox-sensitivity and FA-receptor recognition, which formed by **FA**-DEX-***ss***-**P**TX and **A**DD were referred to as FA-*ss*-P/A. PTX-loaded micelles, featuring redox-sensitivity and FA-receptor recognition, were formed by **FA**-DEX-***ss***-**P**TX, and are referred to as FA-*ss*-P. The PTX and ADD co-loaded micelle with redox-insensitive and FA-receptor recognition, formed by **FA**-DEX-***cc***-**P**TX and **A**DD were denoted as FA-*cc*-P/A. Finally, DEX-***ss***-**P**TX and **A**DD formed micelles with redox-sensitive, but without FA-receptor recognition, were named as *ss*-P/A. The composition, and names, of these different drug-loaded micelles are shown in Supplementary Table S1. Size distribution and topography were determined by dynamic light scattering (DLS) and transmission electron microscopy (TEM), respectively, and the physicochemical characteristics of all micelles are summarized in Supplementary Table S2. TEM showed that the FA-*ss*-P/A micelles were spherical in shape and that size was approximately uniform (Figure 2(A)). The DLS assay showed that the mean sizes of FA-*ss*-P/A, FA-*ss*-P, FA-*cc*-P/A, and *ss*-P/A micelles were 76 ± 2 nm, 67 ± 2 nm, 63 ± 5 nm, and 79 ± 4 nm, respectively. Zeta potential was −11.2 ± 0.8, −12.4 ± 1.1, −11.7 ± 0.9, and −8.5 ± 0.7, respectively (Supplementary Table S2). The CMC of FA-DEX-*ss*-PTX, FA-DEX-*cc*-PTX, and DEX-*ss*-PTX, was 3.1, 4.7, and 5.9 µg/mL (Figure 2(B–D)), respectively. The negative surface potential and low CMC ensured that these micelles remained stable under systemic circulation and hemodilution (Wang et al., 2016). To investigate the stability of these micelles under storage and blood circulation conditions, we incubated freshly prepared micelles in PBS with or without 10% FBS. As shown in Figure 2(E,F), none of the micelles showed any significant change in size during the incubation period, indicating that these micelles could maintain stability under storage and blood circulation conditions. Moreover, the PTX loading efficiency of FA-*ss*-P/A, FA-*ss*-P, FA-*cc*-P/A, and *ss*-P/A, micelles were determined to be 21.3 ± 1.4%, 23.2 ± 1.1%, 19.6 ± 2.1%, and 26.4 ± 1.4% (Supplementary Table S2).

### 3.3. GSH-triggered disassembly and drug release

**Figure 3.**
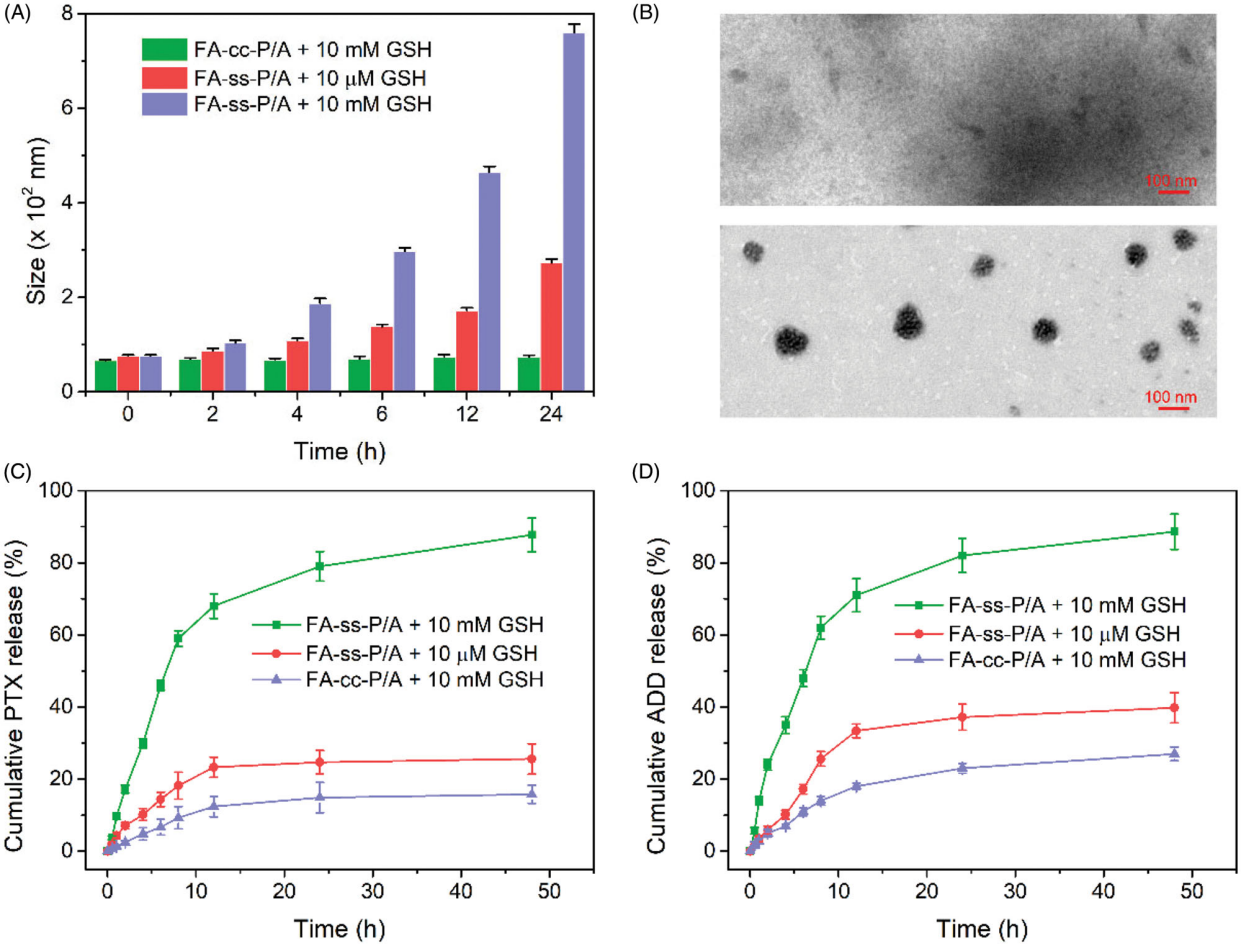
(A) Size changes of FA-*ss*-P/A and FA-*cc*-P/A after treatment with GSH. Data are shown as mean ± SD, *n* = 3. (B) TEM images of FA-*ss*-P/A, and FA-*cc*-P/A, after incubation with 10 mM GSH for 24 h. (C, D) Cumulative release of PTX (C), and ADD (D), from FA-*ss*-P/A and FA-*cc*-P/A in different conditions. Data are shown as mean ± SD, *n* = 3.

The disulfide bond between PTX and FA-DEX can respond to redox signals and release an active form of PTX via a self-immolation pathway (Supplementary Figure S7), resulting in the disassembly of the micelles. To investigate the redox-response-mediated disassembly of the micelles, we used TEM and DLS to determine the morphology and size change of FA-*ss*-P/A and FA-*cc*-P/A under different GSH conditions. As shown in Figure 3(A), the size of the FA-*ss*-P/A micelles changed from 75.8 ± 2.2 nm to 272.5 ± 8.7 nm, and to 759.3 ± 18.7 nm, after 24 h incubation in 10 µM and 10 mM GSH conditions, respectively. In contrast, the size of the FA-*cc*-P/A micelles showed no significant change in size after culture in 10 mM GSH for 24 h. Morphological observations were consistent with the DLS results (Figure 3(B)), demonstrating that the FA-*ss*-P/A micelles were highly sensitive to GSH.

We also used dialysis methods to study the pattern of drug release triggered by GSH. As shown in Figure 3(C), following incubation with 10 µM and 10 mM GSH for 48 h, the cumulative release of PTX from FA-*ss*-P/A micelles was 25.6 ± 4.2% and 87.7 ± 4.7%, respectively. Data also showed that 39.8 ± 3.9% and 88.6 ± 5.1% ADD was released from the FA-*ss*-P/A micelles after treatment with 10 µM and 10 mM GSH for 48 h (Figure 3(D)). However, the cumulative release of PTX and ADD from FA-*cc*-P/A micelles was <30% following culture in 10 mM GSH for 48 h. This result further confirmed the high sensitivity of FA-*ss*-P/A micelles to GSH.

### 3.4. FA-enhanced cell uptake

**Figure 4.**
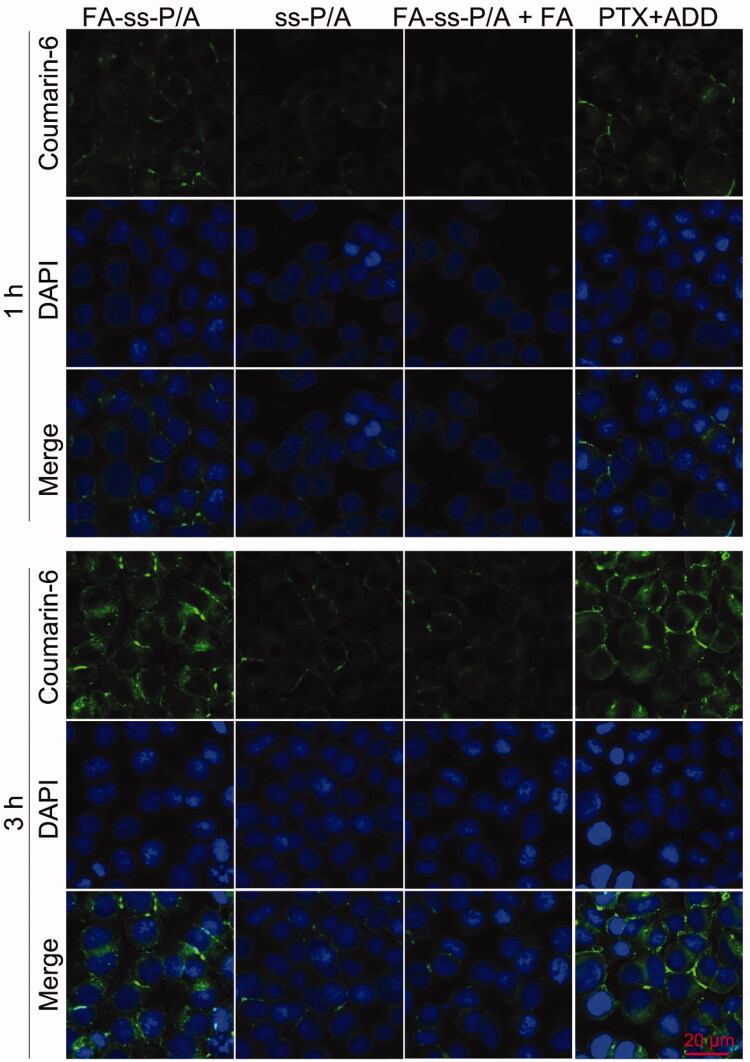
CLSM images of HCT-8/PTX cells treated with FA-*ss*-P/A, *ss*-P/A, FA-*ss*-P/A + FA, or PTX ± ADD for 1 h or 3 h, respectively.

To investigate the targeting ability of FA-*ss*-P/A micelles against HCT-8/PTX cells overexpressing the FA-receptor, we used CLSM and FCM to observe cell uptake behavior. As shown in Figure 4, the fluorescence intensity of both the FA-*ss*-P/A and *ss*-P/A treatment groups increased when the incubation time was extended from 1 h to 3 h, thus confirming the time-dependence of cellular uptake. Moreover, the fluorescence intensity of the FA-*ss*-P/A group was stronger than that of the *ss*-P/A group after the same period of incubation, indicating the high uptake of FA-*ss*-P/A micelles by HCT-8/PTX cells. However, when the cells were pretreated with free FA, the fluorescence in the FA-*ss*-P/A and *ss*-P/A groups showed no significant difference, and the fluorescence signal in the FA-*ss*-P/A group was remarkably weaker than that without FA pretreatment. These results confirmed that the modified FA on the surface of micelles could increase the levels of cellular uptake. Moreover, free drug-treated cells were also showed a high fluorescence intensity, due to the small molecule drugs can quickly diffuse into cells in vitro. We also used FCM to quantitatively analyze the cellular uptake behavior. As shown in Supplementary Figure S8, after 1 h, 3 h and 6 h of treatment, the mean fluorescence intensity (MFI) in the FA-*ss*-P/A group was 1.8-, 1.9-, and 2.8-fold higher than that of the *ss*-P/A group. In contrast, following FA pretreatment, the MFI in the FA-*ss*-P/A group and the *ss*-P/A group was not significantly different. These results further demonstrated that FA-*ss*-P/A was associated with remarkably improved levels of cellular uptake.

### 3.5. PTX efflux was effectively inhibited by ADD

We hypothesized that the incorporation of FA into our drug delivery system would significantly increase drug accumulation in MDR cells, and that the co-delivery of the P-gp inhibitor would reduce drug efflux. The cellular uptake study described above demonstrated that modification of the surface of the micelles with FA enhanced their internalization by HCT-8/PTX cells, resulting in improved levels of intracellular drug accumulation. We therefore analyzed the ability of ADD to reduce the levels of drug efflux, as mediated by P-gp.

Prior to the drug efflux assay, we determined the expression levels of P-gp in both cell lines by western blotting. As shown in Supplementary Figure S9, P-gp was overexpressed in HCT-8/PTX cells at a level that was 8.1-fold higher than that in HCT-8 cells. This result was consistent with a previous report (Jemal et al., 2010). Thus, our results indicate that HCT-8/PTX cells showed strong resistance to multiple drugs, and that HCT-8 cells were relatively sensitive.

**Figure 5.**
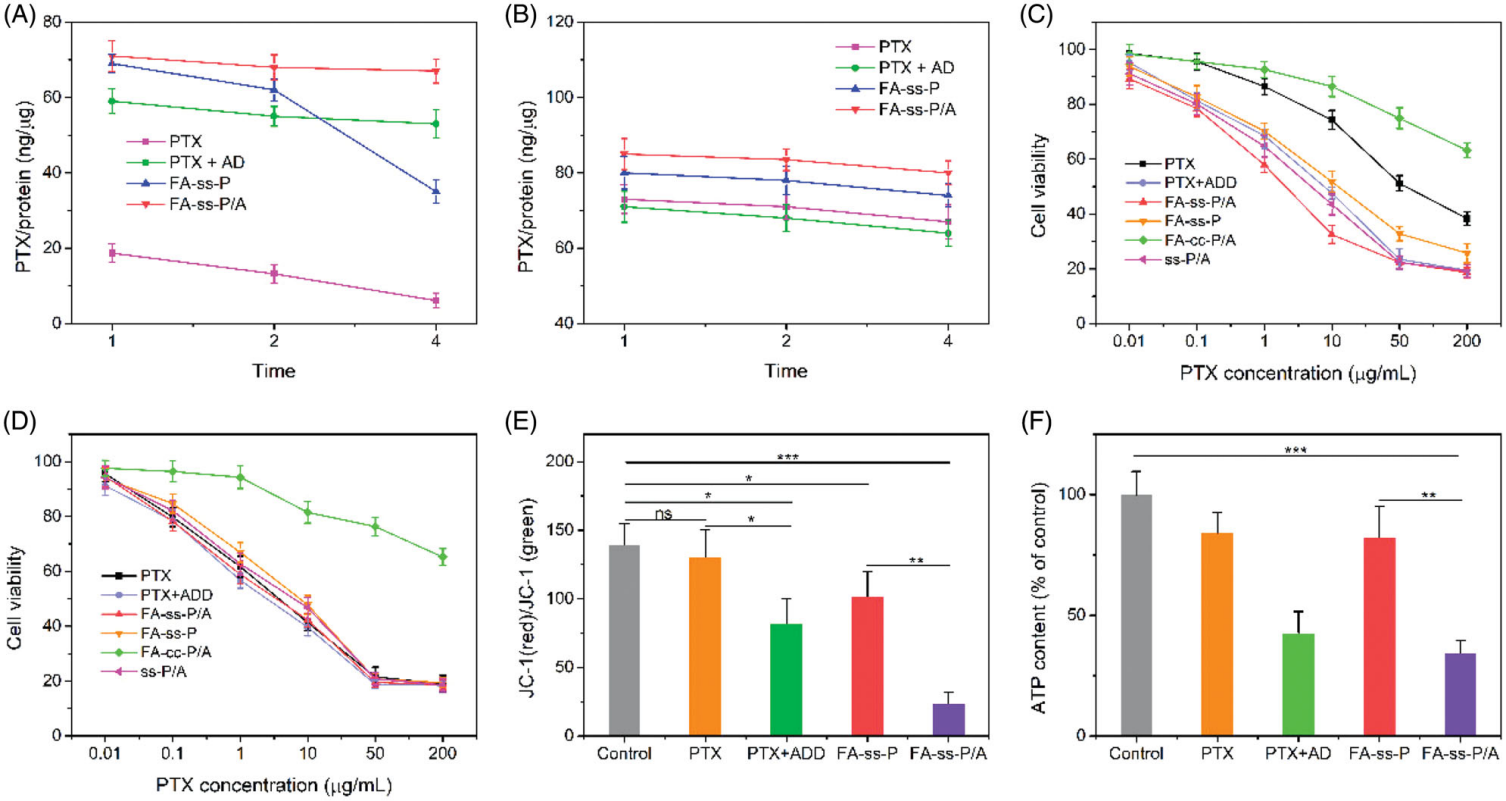
(A, B) The efflux of PTX from HCT-8 cells (A) and HCT-8/PTX cells (B). Cells were first treated with PTX, PTX + ADD, FA-*ss*-P/A, and FA-*ss*-P, for 4 h, and then cultured in fresh medium for 1, 2, or 4 h. Data are shown as mean ± SD, *n* = 6. (C, D) *In vitro* cytotoxicity of PTX, PTX + ADD, FA-*ss*-P/A, FA-*ss*-P, FA-*cc*-P/A, and *ss*-P/A, against HCT-8/PTX cells (C) and HCT-8 cells (D). Data shown as mean ± SD, *n* = 6. (E) Mitochondrial membrane potential of HCT-8/PTX cells after treatment with different drug formulations. (F) Intracellular ATP level in HCT-8/PTX cells after incubation with different drug formulations. Data shown as mean ± SD, *n* = 6. *p < .5, **p < .01, ***p < .001.

To investigate drug efflux, we first cultured cells with PTX, PTX + ADD, FA-*ss*-P, or FA-*ss*-P/A, for 4 h; this was followed by incubation in a drug-free medium for different time period. As shown in Figure 5(A), in drug-sensitive HCT-8 cells, the intracellular level of PTX level (for all drug formulations) slowly decreased when time was extended from 1 h to 4 h. There was no significant difference in intracellular concentration when compared across the different treatment groups. In contrast, in PTX-resistance HCT-8/PTX cells (Figure 5(B)), the intracellular levels of PTX in the free-PTX treatment group were very low and continuously decreased within subsequent incubation; this was due to the efflux effect induced by P-gp. When PTX was combined with ADD, we found that the intracellular level of PTX was significantly higher, and showed no obvious reduction. Similarly, the intracellular levels of PTX in HCT-8/PTX cells decreased with increasing treatment time with FA-*ss*-P micelles; this was due to PTX being pumped out of the cells by P-gp after release from the micelles. However, when HCT-8/PTX cells were treated with FA-*ss*-P/A, the highest levels of PTX in cells can be observed and no apparent reduction, due to the FA-mediated cellular uptake enhancement and the ADD-mediated P-gp action inhibitory.

### 3.6. *In vitro* antitumor efficacy

The experiments described above demonstrated that FA-*ss*-P/A micelles enhance cellular uptake, increase drug accumulation, and reduce the amount of PTX pumped out of HCT-8/PTX cells. Therefore, the FA-*ss*-P/A micelles may have significant potential to overcome MDR. To investigate the ability of these micelles to reverse MDR, we used MTT assays to evaluate the inhibition of cell growth by different drug formulations. As shown in Figure 5(C,D) and Supplementary Table S3, free-PTX exhibited remarkable cytotoxicity in HCT-8 cells and weak cytotoxicity in HCT-8/PTX cells, with approximately 50% cell viability, after treatment with 50 μg/mL of PTX for 48 h. When combined with ADD, the level of growth inhibition was slightly increased in HCT-8 cells; this was because of the synergistic effects of PTX and ADD. However, the cytotoxicity of the PTX + ADD group against HCT-8/PTX cells was significantly higher than that of free-PTX; the IC_50_ value of the PTX + ADD group was 7.9-fold lower than that of PTX in HCT-8/PTX cells. In comparison with control micelles, FA-*ss*-P/A micelles exhibited the highest cytotoxicity against both cell lines; this was due to enhanced cellular uptake, reduced P-gp-mediated drug efflux, and rapid intracellular drug release. Moreover, in both cell lines, the growth inhibition of FA-*ss*-P/A was higher than that of FA-*ss*-P and *ss*-P/A owing to the synergistic effects of PTX and ADD and because FA improved cellular uptake. In addition, the cytotoxicity of the FA-*cc*-P/A group was significantly lower than the FA-*ss*-P/A group in both cell lines; this was because of the uncleavable linkage between PTX and FA-DEX in FA-*cc*-P/A, which resulted in a reduced level of drug release.

### 3.7. The influence of ADD on mitochondrial membrane potential and intracellular ATP content

Previous research highlighted that ADD is a mitochondrial inhibitor that can diminish ATP supply and mediate apoptosis by disrupting mitochondrial function; consequently reducing the excretion of intracellular chemotherapeutics such as doxorubicin (Li et al., 2015; Qi et al., 2018; Wang et al., 2019). Here, we studied the effect of ADD on mitochondrial function and used the JC-1 method to determine the mitochondrial membrane potential. The changes in mitochondrial membrane potential observed after treating HCT-8/PTX cells with different drug formulations for 8 h are presented in Figure 5(E). The free-PTX and FA-*ss*-P micelles had no obvious influence on mitochondrial membrane potential; this was because of the absence of ADD. In contrast, free ADD could significantly reduce the membrane potential. When PTX was combined with ADD (PTX + ADD group and the FA-*ss*-P/A group), the mitochondrial membrane potential was remarkably reduced. These results demonstrate that ADD and FA-*ss*-P/A could effectively inhibit mitochondrial function.

Moreover, dysfunctional mitochondrial would lead to the reduction of intracellular ATP levels, thus inhibiting the pump-out effect mediated by P-gp. As shown in Figure 5(F), compared with the control group, the free-PTX and FA-*ss*-P treatments had no significant influence on intracellular ATP content. However, when cells were treated with ADD, PTX + ADD, and FA-*ss*-P/A, the intracellular ATP content was significantly lower than in the control group. This reduction in intracellular ATP was in accordance with the loss of mitochondrial membrane potential. These results demonstrated that ADD improved the cytotoxicity of MDR cells to PTX by reducing mitochondrial membrane potential and reducing the levels of ATP.

### 3.8. Pharmacokinetics and biodistribution

Because the drug-loaded micelles were administered by *i.v.* injection, it is imperative that they are stable and hemocompatible. Prior to *in vivo* experiments, we investigated the hemocompatibility of all drug-loaded micelles using a hemolysis assay. As shown in Supplementary Figure S10, the rate of hemolysis rate increased as the concentration of micelles increased, and the rate of hemolysis (for all drug-loaded micelles) remained lower than 5% as the concentration of PTX rose from 0.5 µg/mL to 200 µg/mL. According to the standards recommended by the American Society for Testing and Materials (ASTM E2524-08), a hemolysis rate lower than 5% indicates good levels of hemocompatibility (Lucky et al., 2016; Abelha et al., 2019). These data demonstrate that the micelles have high levels of hemocompatibility.

Next, we investigated the *in vivo* pharmacokinetics of PTX and FA-*ss*-P/A (6 mg PTX/kg) by injecting these two drugs (*i.v*.) into HCT-8/PTX tumor-bearing mice. We then used HPLC to detect the plasma levels of PTX at different time points (Supplementary Figure S11(A)). In comparison with free PTX, the FA-*ss*-P/A showed a prolonged circulation time with an elimination half-life of 9.61 ± 0.71 h; this was 3.8-fold higher than that of free-PTX. Furthermore, we also investigated the biodistribution of PTX and drug-loaded micelles following injection (*i.v*.) into HCT-8/PTX tumor-bearing mice. As shown in Supplementary Figure S11(B), in comparison with free PTX and *ss*-P/A, after 24 h, the FA-*ss*-P/A showed a 7.6- and 5.4-fold increase in drug concentration in tumors. These results suggest that FA-*ss*-P/A had a high tumor-targeting efficiency due to FA-mediated active targeting and enhanced permeability and retention (EPR) effect based passive targeting effects.

### 3.9. *In vivo* anti-tumor effect

**Figure 6.**
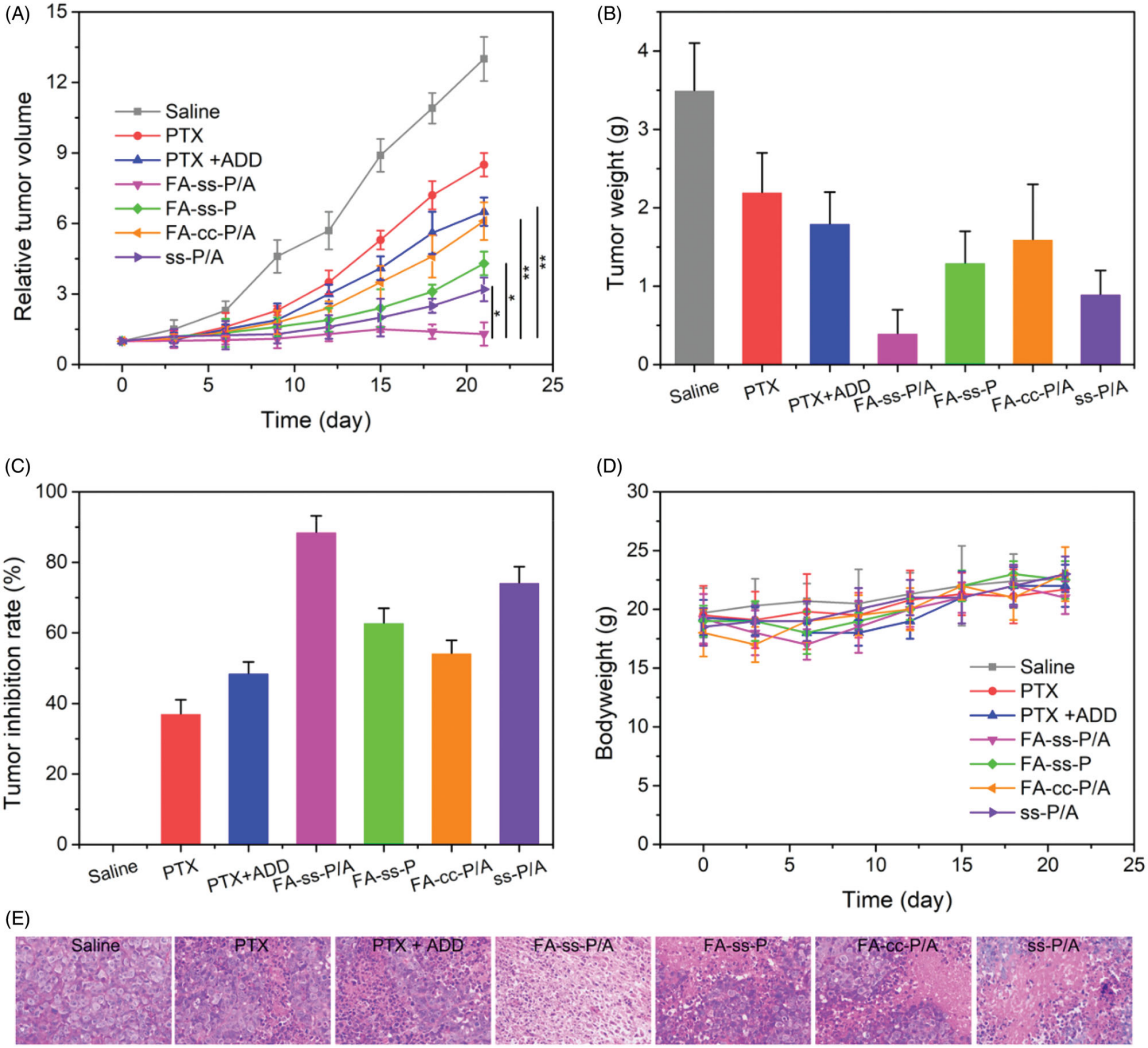
*In vivo* antitumor effect of different drug formulations. (A) Tumor volume over the period of treatment. (B) Tumor weight on day 21 when the mice were sacrificed. (C) Tumor inhibition rate of different drug formulations. (D) Body weight changes during the period of treatment. (E) Hematoxylin and eosin staining of tumor tissue in different drug-treatment groups. Data are shown as mean ± SD, *n* = 5.

To investigate the potential of the micelles to overcome MDR, we next investigated the effect of FA-*ss*-P/A on HCT-8/PTX tumor-bearing mice. As shown in Figure 6(A–C), all drug formulation groups exhibited an obvious suppression of tumor growth at day 21 in compared with that in a saline-administered group; the strongest inhibition was observed with FA-*ss*-P/A. The tumor inhibitory rate (TRI) for FA-*ss*-P/A was 1.6-fold higher than that of the FA-*cc*-P/A group; this was mainly attributed to the complete intracellular drug release of FA-*ss*-P/A in response to GSH. Note that a significant difference was observed in terms of TRI between FA-*ss*-P/A and *ss*-P/A, thus indicating that active tumor-targeting could remarkably enhance antitumor activity. In comparison, the FA-*ss*-P/A group exhibited notably better inhibitory effects than the FA-*ss*-P treated group, suggesting that the combination of PTX and ADD exhibited excellent ability to overcome MDR. These results demonstrate that the strategy of combining active tumor-targeting, the co-delivery of an antitumor drug and a chemo-sensitive drug, and efficient intracellular drug release could effectively combat MDR. To further evaluate the treatment effect of FA-*ss*-P/A, we stained tumor tissue from mice with hematoxylin and eosin. As shown in Figure 6(E), we observed greater remission of tumor cells in the FA-*ss*-P/A treatment group compared with that achieved using other formulations. This provided further evidence of the superior therapeutic efficacy of FA-*ss*-P/A.

Furthermore, we studied the systemic toxicity of all drug formulations by evaluating body weight as an indicator of systemic toxicity. As shown in Figure 6(D), there were no significant differences in body weight for any of the drug treatments over the period of examination, indicating that these drug formulations showed good levels of safety.

## 4. Conclusions

In summary, we constructed active-targeting and redox-responsive micelles to co-deliver PTX and ADD in order to overcome MDR. The micelles were selectively internalized by tumor cells through receptor-mediated endocytosis, and drug release was observed to occur in a controlled manner over time in response to the GSH-rich environment. Most importantly, the combination of active targeting and the inhibition of mitochondrial function by ADD led to high levels of PTX accumulation in drug-resistant tumor cells. Therefore, the present micelle system exhibits significant capacity to suppress MDR tumors *in vivo*. In this study, we created a new drug delivery system with the potential to treat drug-resistant tumors.

## Supplementary Material

Supplemental Material
