# Supplementary material for: Adjudin-loaded redox-sensitive paclitaxel-prodrug micelles for overcoming multidrug resistance with efficient targeted Colon cancer therapy
Source: Drug Deliv. 2020 Jul 24;27(1):1094–105. doi: 10.1080/10717544.2020.1797245 (PMC7470106; doi:10.1080/10717544.2020.1797245)
Supplement: Supplemental Material [file IDRD_A_1797245_SM2242.docx]

**Adjudin loaded redox-sensitive paclitaxel-prodrug micelles for overcoming multidrug resistance with efficient targeted colon cancer therapy**

Deli Chen, Sitang Ge, Lugen Zuo, Shuanhu Wang, Mulin Liu, Shiqing Li

Department of Gastrointestinal Surgery, The First Affiliated Hospital of Bengbu Medical College, Bengbu 233004, China

Corresponding author:

Shiqing Li, Department of Gastrointestinal Surgery, The First Affiliated Hospital of Bengbu Medical College, Bengbu 233004, China

E-mail: l[ishiqing8769@sina.cn](mailto:ishiqing8769@sina.cn)

**Supporting materials and experiments**

**1. Materials**

Dextran (DEX, MW 10 kDa) and paclitaxel (PTX) were purchased from Energy Chemical (Shanghai, China). 3,3-dithiodipropionic acid (DTPA), folic acid (FA), 1-(3-dimethylaminopropyl)-3-ethylcarbodiimide hydrochloride (EDC), 4-Dimethylaminopyridine (DMAP), glutathione (GSH), and glutaric anhydride (GA) were purchased form Aladdin Reagent Company (Shanghai, China). Adjudin (ADD) was obtained from Bide Pharmaceutical Technology Co., Ltd. (Shanghai, China). DAPI, 3-(4,5-dimethylthiazol-2-yl)-2,5-diphenyl tetrazolium bromide (MTT), Triton X-100, and BCA kit were purchased from Beyotime Institute of Biotechnology (Shanghai, China).

**2. Instrumentation**

^1^H NMR spectra were recorded on a Varian U500 (300 MHz) spectrometer. Particle size and polydispersities (PDI) were determined by a dynamic light scattering (DLS, ZetaPlus, USA). The morphology was observed by transmission electron microscopy (TEM, JEM, Japan). High-performance liquid chromatography (HPLC) was performed on a Shimadzu HPLC system (LC-20A). Shimadzu C18 column (5 µm, 250 mm × 4.6 mm) was used for analysis. For PTX analysis, the mobile phase: methanol/water = 60/40, v/v; the detection wavelength set as 227 nm. For ADD analysis, the mobile phase: methanol/water = 87/13, v/v, with 1% anhydrous acetic acid, and the detection wavelength set as 302 nm.

**3. Stability of drug loaded micelle**

The stability of all micelles at different conditions were evaluated. The drug loaded micelles were dispersed in PBS with or without 20% fetal bovine serum (FBS) at the final concentration of drug loaded micelles was 6 mg/mL. Subsequently, these mixtures were incubated at 4 °C or 37 °C for different times, at the intervals time, the size of these micelles were determined by DLS.

**4. Cell culture and animals**

The human colon cancer cell line HCT-8 and HCT-8/PTX cells were purchased from KeyGEN BioTECH (Nanjing, China). These cell lines were cultured in RPMI 1640 supplemented with 10% fetal bovine serum (FBS) and 100 IU/mL penicillin and 100 µg/mL streptomycin at 37 °C in a humidified 5% CO_2_ atmosphere. Drug resistance of HCT-8/PTX was maintained by the addition of PTX (5 µg/mL) in the medium.

BALB/c nude mice (female, 4-6 weeks, 20 ± 2 g) were purchased from the Vital River Laboratory Animal Technology Co., Ltd. (Beijing, China). All animals received care in compliance with the guidelines outlined in the Guide for the Care and Use of Laboratory Animals and all procedures were approved by The Bengbu Medical College.

**5. Hemolysis analysis**

The hemocompatibility of all micelles at different concentrations was analyzed by hemolysis assay. Typically, fresh mice blood was diluted by saline, and red blood cells (RBCs) were collected by centrifugation. Subsequently, the RBCs were further diluted by saline. The suspension obtained (2%) was used for hemolysis study. Drug loaded micelles at different concentrations (equal to PTX concentration as 0.5, 1.0, 5.0, 10.0, 50.0, 100.0, and 200.0 μg/mL) were added in RBCs suspension and incubated for 2 h at 37 °C. After that, the mixtures were centrifuged at 1200 rpm for 10 min. The supernatant was collected and the amount of hemoglobin released was recorded on a microplate reader at 540 nm. Water was used as positive control and PBS was used as a negative control. The hemolysis ratio (HR) of RBCs was calculated according to the following formula:

Hemolysis (%) = (As – An) / (Ap – An) × 100, where, “As” is the absorbance of sample, “An” is the absorbance of negative control and “Ap” is the absorbance of positive control. All hemolysis experiments were carried out in triplicate.

**6. Statistical analysis**

All the data were recorded using means ± standard division (SD). Statistical differences were determined using student’s T-test or one-way ANOVA. P < 0.05 was considered to be significant difference and all the data were measured in triplicate.

**Support Figures and Table:**


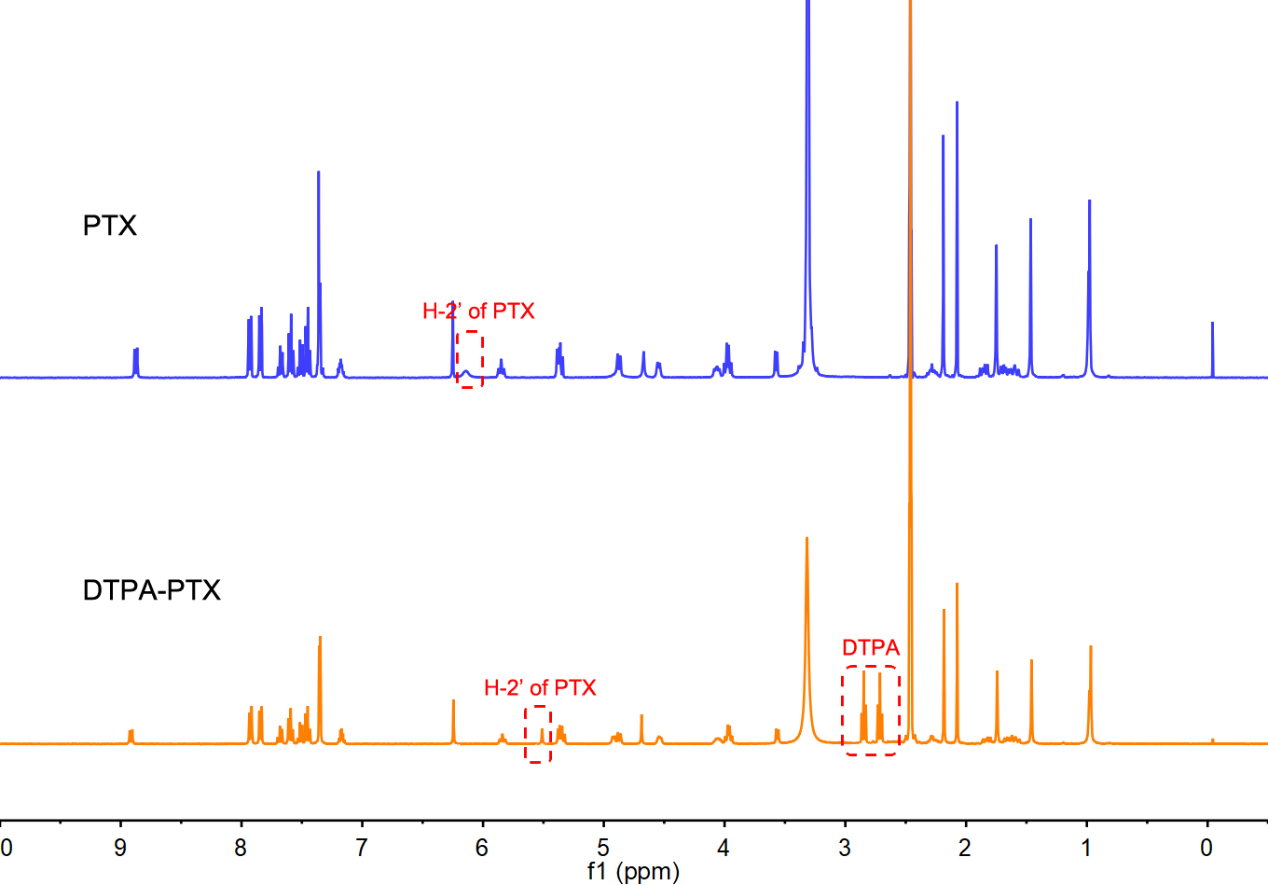


**Fig. S1** ^1^H NMR spectrum of PTX and DTPA-PTX in DMSO-d6.





**Fig. S2** Mass spectrum of DTPA-PTX.


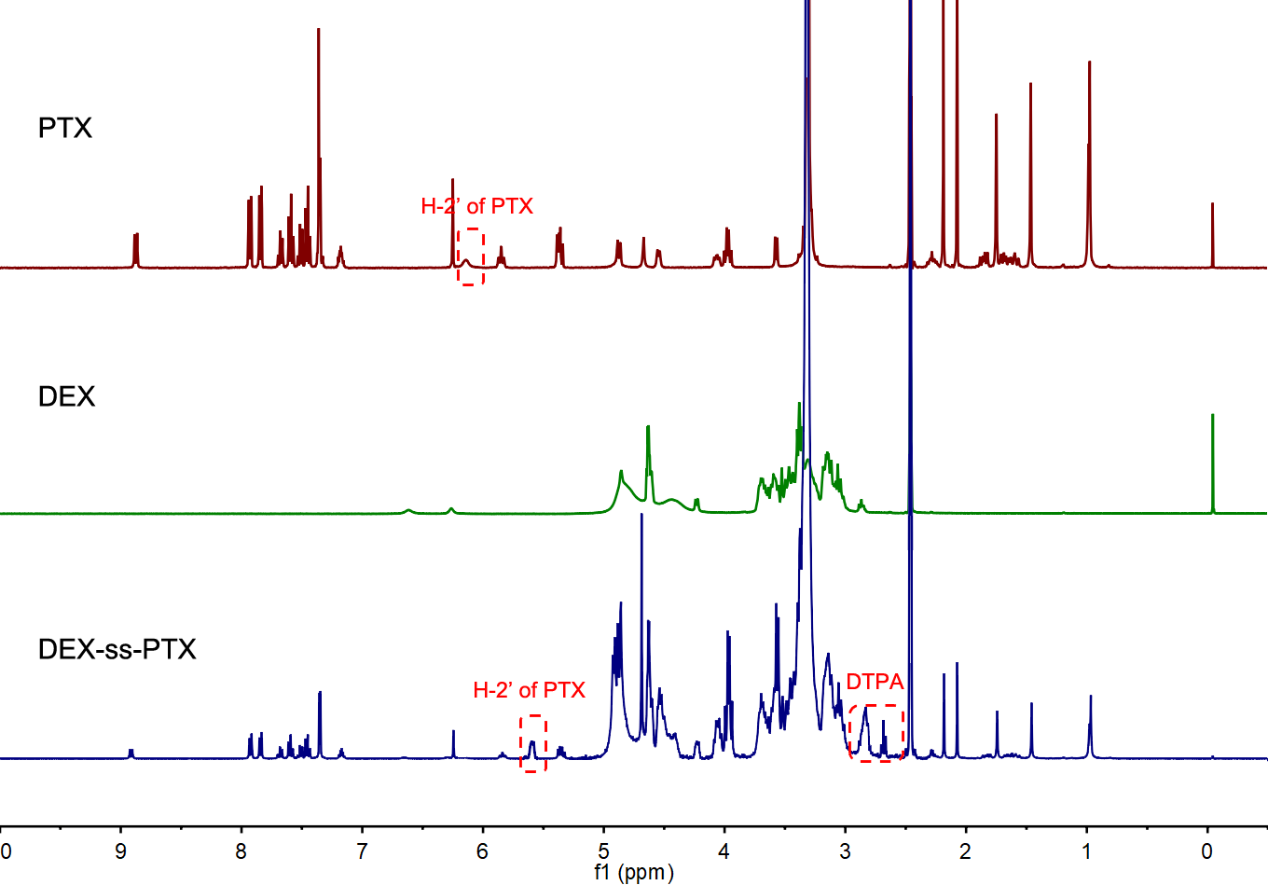


**Fig. S3** ^1^H NMR spectrum of PTX, DEX, and DEX-ss-PTX in DMSO-d6.


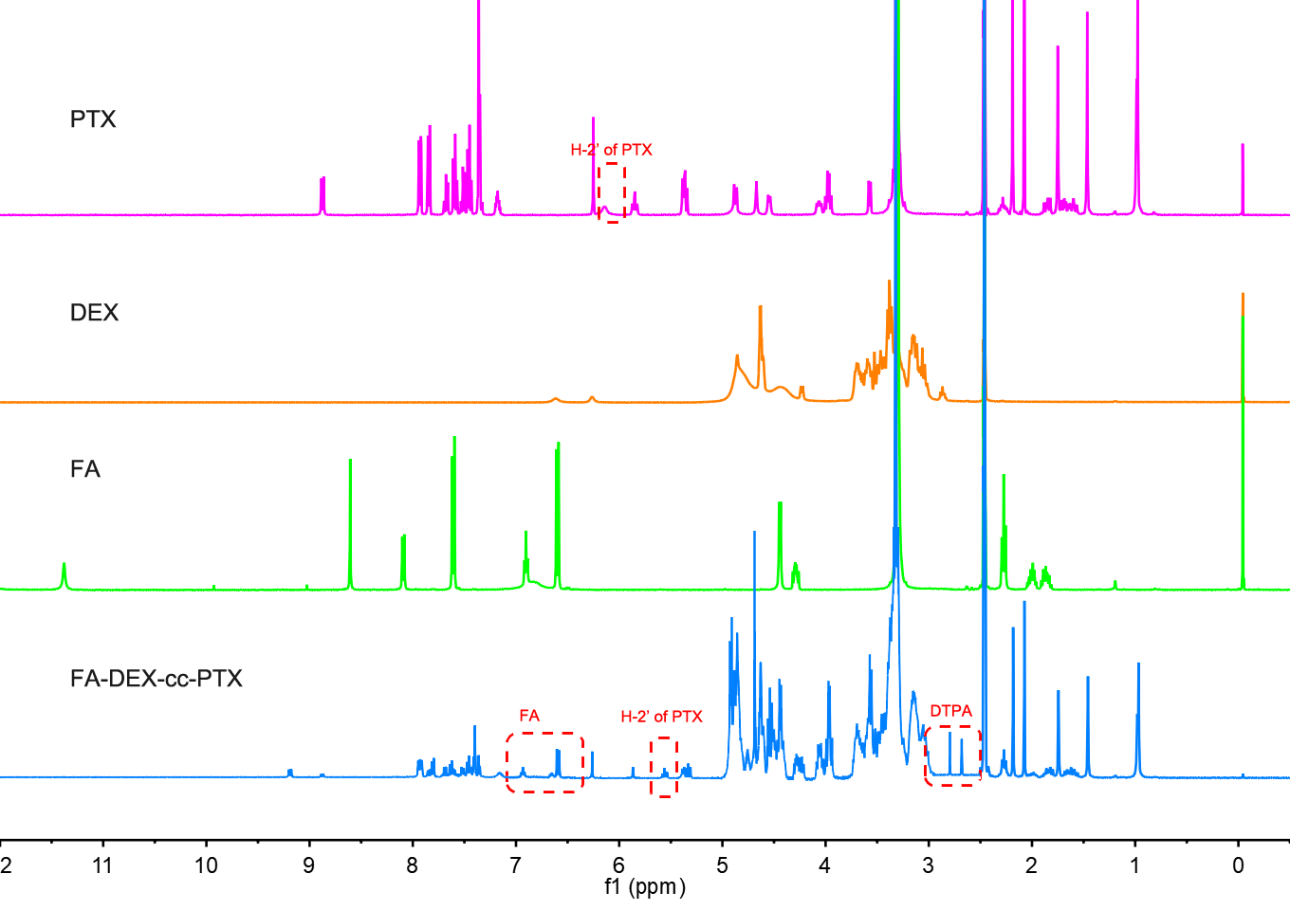


**Fig. S4** ^1^H NMR spectrum of PTX, DEX, FA, and FA-DEX-cc-PTX in DMSO-d6.





**Fig.S5** HPLC analysis of PTX, FA-DEX-ss-PTX, and GSH treatment of 2 h FA-DEX-ss-PTX.





**Fig. S6** The IC50 value of PTX/ADD at different ratio in against HCT-8/PTX cells.





**Fig. S7** PTX release mechanism.





**Fig. S8** HCT-8/PTX cells uptake of FA-ss-P/A, FA-ss-P, or FA-ss-P/A + HA at different incubation time. Data showed as mean ± SD, *n* = 6.


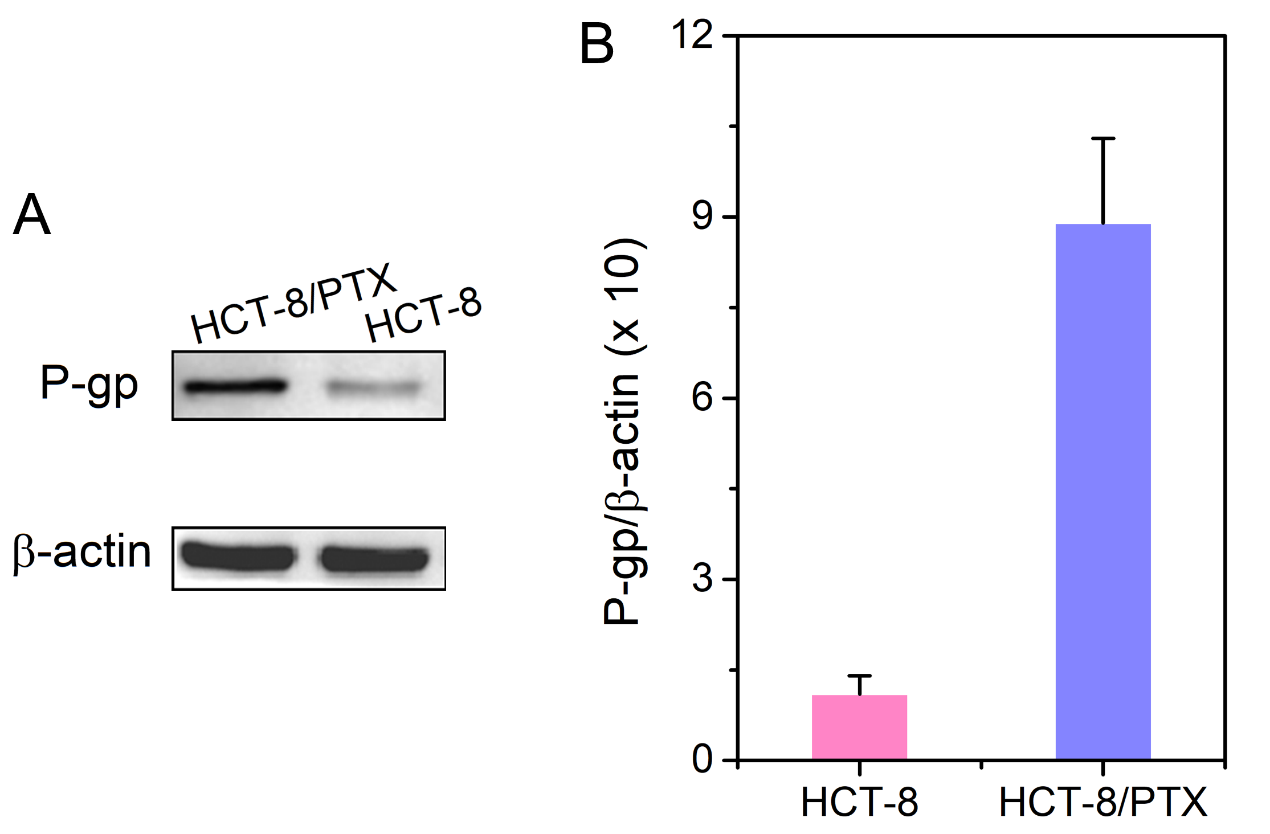


**Fig. S9** P-gp expression level in HCT-8 cells and HCT-8/PTX cells. (A) Western blotting images. (B) Quantitative result of western blotting. Data showed as mean ± SD, *n* = 3.





**Fig. S10** Hemolysis analysis of FA-ss-P/A, FA-ss-P, FA-cc-P/A, and ss-P/A micelles. Data are shown as mean ± SD, *n* = 3.


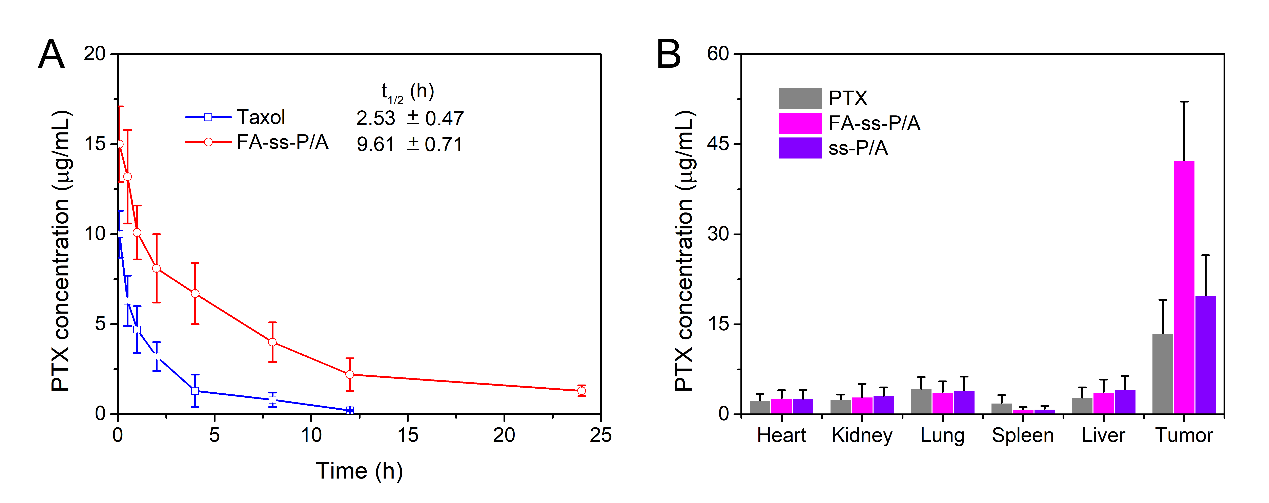


**Fig. S11** (A) *In vivo* pharmacokinetics of PTX and FA-ss-P/A. Data are shown as mean ± SD, *n* = 6. (B) *In vivo* biodistribution of PTX, FA-ss-P/A, and ss-P/A, in HCT-8/PTX tumor bearing mice. Data are shown as mean ± SD, *n* = 6.

| **Table S1.** The composition of different drug loaded micelles | | | | |
| --- | --- | --- | --- | --- |
| Micelles | FA-DEX-ss-PTX | FA-DEX-cc-PTX | DEX-ss-PTX | ADD |
| FA-ss-P/A | + | - | - | + |
| FA-ss-P | + | - | - | - |
| FA-cc-P/A | - | + | - | + |
| ss-P/A | - | - | + | + |

| **Table S2.** Characterization of all drug-loaded micelles | | | | | | |
| --- | --- | --- | --- | --- | --- | --- |
| Micelles | Size (nm) | PDI | Zeta (mV) | DLC (PTX, %) | DLC (ADD, %) | DEE (ADD, %) |
| FA-ss-P/A | 76 ± 2 | 0.24 ± 0.02 | -11.2 ± 0.8 | 21.3 ± 1.4 | 17.6 ± 1.7 | 76.5 ± 6.3 |
| FA-ss-P | 67 ± 2 | 0.21 ± 0.01 | -12.4 ± 1.1 | 23.2 ± 1.1 | 19.4 ± 1.3 | - |
| FA-cc-P/A | 63 ± 5 | 0.18 ± 0.02 | -11.7 ± 0.9 | 19.6 ± 2.1 | 16.1 ± 1.4 | 77.3 ± 4.9 |
| ss-P/A | 79 ± 4 | 0.25 ± 0.01 | -8.5 ± 0.7 | 26.4 ± 1.4 | 21.7 ± 1.6 | 81.5 ± 3.2 |

| **Table S3.** IC50 value of drugs. | | |
| --- | --- | --- |
| IC50 vale (μg/mL) | HCT-8/PTX cells | HCT-8 cells |
| PTX | 59.5 | 2.0 |
| PTX + ADD | 7.5 | 1.6 |
| FA-ss-P/A | 2.1 | 1.7 |
| FA-ss-P | 8.3 | 5.4 |
| FA-cc-P/A | ~ | ~ |
| ss-P/A | 6.4 | 5.0 |
